# Severe Coronary Artery Vasospasm after Mitral Valve Replacement in a Diabetic Patient with Previous Stent Implantation: A Case Report

**DOI:** 10.2478/jccm-2022-0005

**Published:** 2022-05-12

**Authors:** Alexandra Iulia Stoica, Marius Harpa, Cosmin Marian Banceu, Judith Kovacs, Horatiu Suciu

**Affiliations:** 1 George Emil Palade University of Medicine, Pharmacy, Science, and Technology of Targu Mures, Targu Mures, Romania; 2 Institute for Cardiovascular Diseases and Heart Transplantation, Targu Mures, Romania

**Keywords:** postoperative coronary artery vasospasm, mitral valve replacement, venous-arterial extracorporeal membrane oxygenation, hemodynamic instability, drug-eluting stent

## Abstract

Postoperative coronary vasospasm is a well-known cause of angina that may lead to myocardial infarction if not treated promptly. We report a case of a 70-year-old female with severe mitral regurgitation submitted to mitral valve replacement, and a history of diabetes mellitus type II, stroke, idiopathic thrombocytopenic purpura on steroid therapy, and previous percutaneous coronary intervention (PCI) for severe obstruction of the circumflex coronary artery, 4 months prior to surgery. Immediately after intensive care unit admission, the patient developed pulseless electrical activity which required extracorporeal membrane oxygenation for hemodynamic support. The coronary angiography showed diffuse occlusive coronary artery vasospasm, ameliorated after intra-coronary administration of nitroglycerin. The following postoperative evolution was marked by cardiogenic shock and multiple organ dysfunction syndrome. Subsequent echocardiographic findings showed an increase in left ventricular function with an EF of 40%, and extracorporeal membrane oxygenation (ECMO) support was weaned after seven days. However, after a few hours, the patient progressively deteriorated, with cardiac arrest and no response to resuscitation maneuvers. Hemodynamic instability following the surgical procedure in a patient with previous PCI associated with an autoimmune disease and diabetes mellitus should raise the suspicion of a coronary artery vasospasm.

## Introduction

Postoperative coronary vasospasm is a well-known cause of angina that may lead to myocardial infarction if not treated promptly [1]. Clinical manifestations of vasospasm are unpredictable, ranging from electrocardiographic (ECG) changes to severe hemodynamic deterioration [2].

## Case presentation

A 70-year-old female was admitted to the Emergency Institute for Cardiovascular Disease and Transplant of Targu Mures, for surgical treatment of degenerative mitral regurgitation. The patient had a history of diabetes mellitus type II, stroke, idiopathic thrombocytopenic purpura on steroid therapy, and previous percutaneous coronary intervention (PCI) for severe obstruction of the circumflex coronary artery, 4 months prior to surgery.

**Fig. 1:**
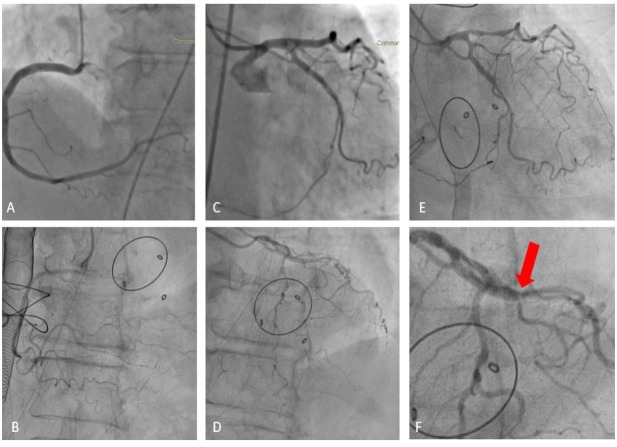
Comparative view of the coronary arteries – preoperative and postoperative aspect (A: Preoperative coronary findings – right coronary artery. B: Postoperative coronary findings – right coronary artery with severe vasospasm. C: Preoperative coronary findings – left anterior descending artery and circumflex artery with permeable DES. D: Postoperative coronary findings – left anterior descending artery and circumflex artery with severe vasospasm. E: Postoperative coronary findings – left anterior descending artery and circumflex artery after administration of Nitroglycerin). F: Stent-edge vasospasm.

Preoperative coronary angiography revealed absence of residual stenosis with normal run-off of the circumflex coronary artery (Fig. 1 – A,C). The electrocardiogram showed atrial fibrillation. Laboratory test results revealed moderate thrombocytopenia.

Transthoracic echocardiography (TTE) showed a severe mitral regurgitation, moderate tricuspid regurgitation, dilation of the left atrium, severe secondary pulmonary hypertension and preserved global contractility with an ejection fraction (EF) of 55%.

The surgical intervention was performed through median sternotomy and aorto-bicaval cardiopulmonary bypass under normothermic conditions. Myocardial protection was obtained using Custodiol cardioplegia.

The mitral valve was replaced with a 27 Medtronic® Hancock biologic prosthesis, with a total ischemia time of 69 minutes. The intraoperative course was uneventful, with hemodynamic stability and preserved cardiac contractility, with minimal inotropic support and an inotropic score of 2.

Approximately one hour after intensive care unit (ICU) admission, the patient developed pulseless electrical activity, therefore resuscitation maneuvers were initiated. Acute causes of hemodynamic instability, such as cardiac tamponade were excluded. The patient was emergently transferred to the operating room. Cardiopulmonary bypass and central venous-arterial extracorporeal membrane oxygenation (V-A ECMO) were initiated, with an inotropic score of 130.

Intraoperative transesophageal echocardiography (TEE) revealed normofunctional biological prosthesis in mitral position and poor left ventricular function. After cardiac rhythm was restored, the patient developed ECG changes consisting of ST elevation, raising suspicion of either acute occlusion of the circumflex artery secondary to the mitral valve replacement, or intrastent occlusion.

The patient was emergently transferred to the catheterization lab and the angiography showed diffuse occlusive coronary artery vasospasm (Fig. 1 – B, D), ameliorated after intra-coronary administration of nitroglycerin (Fig 1 – E).

The following postoperative period in the ICU was marked by significant hemorrhage which required surgical hemostasis. Laboratory test results were modified, with elevated cardiac enzyme levels: troponin (1.744 ng/mL), creatine kinase (709 U/L), creatine kinase-MB (213 U/L), low hemoglobin levels (5.9 g/dL), platelet count (48x10 9/L) and fibrinogen (65.7 mg/dL) as well as high CRP (22 mg/dL) and D-dimers (>1600 ng/mL) levels.

Postoperative evolution was marked by cardiogenic shock and multiple organ dysfunction syndrome. Subsequent echocardiographic findings showed an increase in left ventricular function with an EF of 40%, and ECMO support was weaned after seven days. However, after a few hours, the patient progressively deteriorated, with cardiac arrest and no response to resuscitation maneuvers.

## Discussions

Coronary vasospasm can be a potential cause of acute myocardial infarction, life-threatening arrhythmias and cardiac arrest in patients with or without hemodynamically significant coronary artery disease [3].

The occurrence of vasospasm is frequent after coronary artery bypass [4], and there are a few reports of coronary vasospasm after valve surgery [5, 6].

The most common presentation of coronary vasospasm is in the first 24 hours after surgery.

In some cases, the vasospasm can manifest with ventricular arrhythmias and cardiac arrest, and may require ECMO for hemodynamic support [7].

After initiation of ECMO, the inotropic support might aggravate the coronary vasospasm, therefore it should be discontinued.

Increased levels of endo- or exogenous catecholamines, especially dopamine, may precipitate the vasospasm [8].

In the presented case, V-A ECMO was initiated, but inotropic support could not be discontinued due to persistent hemodynamic instability.

The underlying mechanisms of postoperative coronary artery spasm are not clearly understood. However, several precipitating factors have been identified, including atherosclerosis, sympathetic-adrenergic stimulation, endothelial dysfunction, diabetes mellitus, autoimmune diseases, electrolyte imbalances like magnesium deficiency and hyperkalemia.

Furthermore, the intraoperative factors, such as surgical manipulation or compression caused by drainage tubes, may precipitate the coronary vasospasm as well [9, 10, 11].

According to recent studies, cardiopulmonary bypass (CPB)/Cardioplegia initiates systemic inflammatory response syndrome (SIRS) and is associated with vasomotor dysfunction, which can precipitate coronary artery spasm, these consequences being more pronounced in patients with diabetes mellitus [12].

Chronic inflammation plays a major role in coronary microvascular impairment, high levels of C-reactive protein (CRP) being associated with endothelial dysfunction [13]. Studies have demonstrated that chronic inflammation is associated with coronary microcirculation dysfunction in patients with autoimmune diseases, like lupus erythematosus or rheumatoid arthritis [14, 15].

Moreover, diabetes mellitus is associated with micro-vascular dysfunction of the heart, brain and kidneys, even in the absence of dyslipidemia. High levels of insulin, insulin resistance and chronic hyperglycemia contribute to endothelial dysfunction by promoting an inflammatory response, increased oxidative stress, hypercholesterolemia, and progression of atherosclerosis [16, 17].

Although in the presented case the intraoperative myocardial ischemia time was not increased and the patient was on minimal inotropic support with dopamine, triggers like CPB/cardioplegia and diabetes mellitus might have been factors that contributed to the vasospastic event.

Drug-eluting stents (DES) can lead to an abnormal tendency of vasomotor dysfunction, especially in the presence of endothelial dysfunction. Studies have shown that Rho-kinase pathway plays a role in the pathogenesis of coronary vasospasm. Shiroto et al. have suggested that DES increased Rho-kinase expression and activity, which is involved in coronary artery vasospasm [18]. Shibutani et al. reported a case of in stent thrombosis secondary to stent-edge spasm, which describes the potential role of DES in coronary artery vasospasm [19]. Another study presented a case of a diabetic patient with previous DES implantation, who experienced coronary artery vasospasm after coronary artery bypass grafting with favorable response to intracoronary fasudil injection which is a Rho-kinase inhibitor [20].

In healthy patients, intracoronary infusion of acetylcholine results in coronary vasodilatation; however, in the presence of atherosclerosis, it produces coronary artery spasm. Numerous studies have shown that the presence of DES may cause severe coronary artery spasm at the stent site after intracoronary administration of acetylcholine, which can indicate endothelial dysfunction due to DES implantation [21, 22, 23, 24].

According to a study that assessed the relation between stent implantation and coronary spasm, a significant frequency of vasospasm was found in both infarct related arteries and non-infarct related arteries. In the presented case, the patient had DES implantation 4 months prior to surgery, which could have been a trigger for multivessel coronary spasm [25].

Surgical manipulation of the heart, the use of CPB/ cardioplegia, sympathetic stimulation, and the presence of endothelial dysfunction in a diabetic patient with previous DES implantation may have contributed to the occurrence of coronary artery spasm.

Coronary artery spasm is a multifactorial pathology that can lead to life-threatening consequences, including death, requiring early recognition and treatment, especially in diabetic patients [26].

## Conclusion

The diagnosis of postoperative coronary artery spasm is difficult to establish. Hemodynamic instability following the surgical procedure in a patient with previous PCI associated with an autoimmune disease and diabetes mellitus should raise suspicion and coronary artery vasospasm should be considered.

To summarize, we believe that DES may play a role in coronary artery spasm, especially in the presence of risk factors like diabetes mellitus and preventive treatment and appropriate preoperative management should be provided for these patients.
